# Ordered SnO_2_@C Flake Array as Catalyst Support for Improved Electrocatalytic Activity and Cathode Durability in PEMFCs

**DOI:** 10.3390/nano10122412

**Published:** 2020-12-02

**Authors:** Zhaoyi Yang, Ming Chen, Baizeng Fang, Gaoyang Liu

**Affiliations:** 1 School of Metallurgical and Ecological Engineering, University of Science and Technology Beijing, 30 College Road, Beijing 100083, China; zhaoyiyangustb@163.com (Z.Y.); chenm3@sustech.edu.cn (M.C.); 2 Beijing Key Laboratory for Magneto-Photoelectrical Composite and Interface Science, University of Science and Technology Beijing, 30 College Road, Beijing 100083, China; 3 Department of Chemical and Biological Engineering, University of British Columbia, 2360 East Mall, Vancouver, BC V6T 1Z3, Canada

**Keywords:** SnO_2_@C, ordered flake array, carbon paper, integrated cathode, proton exchange membrane fuel cell, electrocatalytic activity, cathode durability

## Abstract

Pt-SnO_2_@C-ordered flake array was developed on carbon paper (CP) as an integrated cathode for proton exchange membrane fuel cell through a facile hydrothermal method. In the integrated cathode, Pt nanoparticles were deposited uniformly with a small particle size on the SnO_2_@C/CP support. Electrochemical impedance spectroscopy analysis revealed lower impedance in a potential range of 0.3–0.5 V for the ordered electrode structure. An electrochemically active surface area and oxygen reduction peak potential determined by cyclic voltammetry measurement verified the synergistic effect between Pt and SnO_2_, which enhanced the electrochemical catalytic activity. Besides, compared with the commercial carbon-supported Pt catalyst, the as-developed SnO_2_@C/CP-supported Pt catalyst demonstrated better stability, most likely due to the positive interaction between SnO_2_ and the carbon coating layer.

## 1. Introduction

The depletion of fossil-fuel based sources of energy and the rising global concerns with respect to the emission of greenhouse gases are among the key drivers in the current search for alternative and clean sources of energy [1,2,3,4]. Hydrogen-based technologies and proton exchange membrane fuel cells (PEMFCs) have received much attention for both stationary and automotive applications [5,6,7,8]. However, a number of technical issues, such as the use of expensive Pt catalyst, kinetic loss of the cathode electrode and the poor durability with respect to long term operations have hindered the large-scale deployment of PEMFCs [9,10,11]. Among many alternative approaches for the Pt catalysts, alloying with less expensive metals to reduce the Pt content and also increase electrocatalytic activity, and the use of non-precious metal-based catalysts have been widely explored [12,13,14]. However, Pt and Pt-based catalysts still remain the state-of-the-art catalysts available for PEMFCs [15,16]. As well as the activity of catalyst materials, stability/durability issues of materials used in PEMFCs are also critical and need to be improved. The stability challenges include corrosion of carbon-based catalyst support under exposures to high electrochemical potentials [17], which leads to the detachment and loss of the supported Pt-based nanoparticles from the support, and accordingly the decay in the electrocatalytic performance [18].

Recently, some approaches have been reported for the improvement in the stability of catalyst support, such as the replacement of a carbon microporous layer with graphite oxide and the use of alloy catalyst layers without carbon support on the gas diffusion layer [19,20]. In one approach, the Pt catalyst was directly supported on the carbon fibers to form an integrated cathode [21]. However, carbon fibers provide a low specific surface area, resulting in a low electrochemical active area of the supported Pt electrocatalyst. In order to increase the specific surface area of the support and maintain high support stability, many materials, including metal oxides, carbides and high molecular polymers, have been investigated [22,23,24]. A suitable support not only enhances electrochemical stability, but also contributes to the improvement of the catalytic performance of the supported catalyst. For instance, Liu et al. [25] prepared WO_3_-supported Pt catalysts with high electrochemical activity and stability. In addition to the support material, unhindered transmission channels are desirable and decisive for the reduced mass transport loss, improved three-phase reaction zone and enhanced catalytic activity [26,27,28,29,30,31]. A good example of non-carbon-based ordered support materials is the organic self-assembled crystalline whiskers array structure from 3M Company (St. Paul, MN, United States), which grows by a screw-dislocation mechanism when a vacuum deposited organic pigment (CAS #PR149) film is annealed at 250–270 °C. These support structures replace the traditional carbon-based catalyst support, and have shown excellent stability even after exposure to high electrochemical potentials [32].

Among metal oxide-based materials, SnO_2_ with stable electrochemical performance in sulfuric acid system and oxygen-containing conditions was reported to be a good candidate for catalyst support material [33]. In addition, Cognard et al. [34] and Suffner et al. [35] prepared Sb-doped SnO_2_-supported Pt catalysts, which demonstrated better stability than commercial Pt/C in PEMFC. Zhang et al. [36] used SnO_2_ to modify Pt/C, and showed that the interaction of carbon and SnO_2_ also led to improvements in the stability and activity of the catalyst. Growth of SnO_2_ nanosheet arrays on carbon fiber for the enhancement of polysulfides redox in lithium-sulfur batteries has also been demonstrated by Wang et al. [37].

In this work, we report a novel composite catalyst support, SnO_2_@C/CP, which was prepared by depositing SnO_2_@C with an ordered flake array (OFA) on carbon paper (CP) through a facile solvothermal method. The SnO_2_ array structure provides a high specific surface area and stability, as well as adequate pathways for the transport of reactants and products, while the carbon coating layer reduces the contact resistance and provides ample sites for the homogeneous growth of the Pt catalyst. The impact of the ordered nature of the support structure and carbon coating on the performance and stability were analyzed in depth. The structural changes of the Pt-SnO_2_@C/CP and the baseline Pt/C/GDL (gas diffusion layer) and Pt-SnO_2_/CP cathodes after the accelerated durability testing (ADT) experiments were also examined by scanning electron microscopy (SEM) observations. Due to the unique nanostructure, the as-developed electrocatalyst not only demonstrated enhanced electrocatalytic activity, but also improved cathode durability compared with the commercial carbon-supported Pt catalyst. It was found that the synergistic effect between Pt and SnO_2_ was responsible for the enhanced electrocatalytic activity, while the interaction between SnO_2_ and the carbon layer improved the stability.

## 2. Experimental Section

### 2.1. Materials

Hexachloroplatinic acid (H_2_PtCl_6_•6H_2_O, 99.95%), formic acid (HCOOH, 88%) and other chemicals were purchased from Sinopharm Chemical Reagent Beijing Co., Ltd. (Beijing, China). Commercial 40% Pt/C (Johnson Matthey Co., London, UK), CP (Toray, TGP-H-60, Tokyo, Japan) and all chemicals were used as received without any further purification.

**Figure 1:**
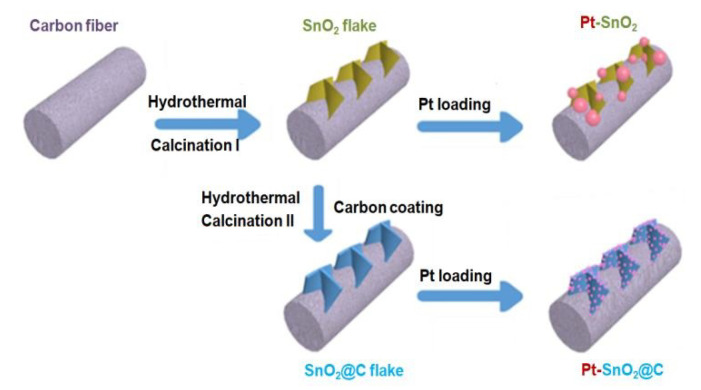
Schematic illustration of the preparation of Pt-SnO_2_/CP and Pt-SnO_2_@C/CP.

Figure 1 presents the schematic illustration of synthesis procedures of Pt-SnO_2_/CP and Pt-SnO_2_@C/CP. First, through a hydrothermal reaction, SnO_2_ grows on the surface of carbon fibre to form a flake-like SnO_2_. Next, a carbon layer is coated on the SnO_2_ to form a core-shell structure. Finally, Pt nanoparticles are deposited on the SnO_2_/CP and SnO_2_@C/CP supports using the same impregnation-reduction method. Details of the syntheses are described in the next section.

### 2.2. Synthesis of SnO_2_/CP OFA

For the hydrothermal method, typically 0.064 g of SnCl_4_ 5H_2_O and 0.064 g of thioacetamide were dissolved in 40 mL of isopropanol [38]. Then, two pieces of CP (5 cm^2^) were placed in a Teflon-lined stainless autoclave (Sh-yantu Co., Shanghai, China) with one side exposed vertically. Next, 40 mL of the prepared solution was transferred into the autoclave and sealed. The reaction was carried out at 180 °C for 24 h. After cooling down to room temperature (20 °C), the two CPs were dried and calcined at 500 °C for 2 h in air to promote the formation of SnO_2_. The flake array samples were denoted as SnO_2_/CP.

### 2.3. Synthesis of SnO_2_@C/CP OFA

To produce the carbon layer, a 4.7 g L^−1^ glucose stock solution was first prepared. Then, about 20 mL of this glucose solution was transferred into the Teflon-lined stainless autoclave [38]. Next, one piece of the as-prepared SnO_2_/CP was placed at the bottom of the reactor ((Sh-yantu Co., Shanghai, China), with the top side (covered with SnO_2_) exposed to the solution, and the reactor was sealed. Later, the reaction vessel was placed in an oven at 180 °C for 24 h. Subsequently, the sample was calcined at 500 °C for 2 h under a flow of argon. This flake sample was denoted as SnO_2_@C/CP.

### 2.4. Deposition of Pt on the Support

Deposition of Pt on the support was achieved via an impregnation-reduction method. About 200 μL of 0.05 M H_2_PtCl_6_ 6H_2_O and 40 μL of 0.5 M HCOOH were dissolved in 4 mL of deionized water as the reaction solution [39,40,41]. A piece of SnO_2_/CP or SnO_2_@C/CP was fixed in a homemade reaction container, and the reaction solution was then poured into the container. The reaction was allowed to proceed at room temperature (20 °C) for 72 h. Finally, the product was washed with deionized water for several times, and dried at 50 °C overnight. The products were denoted as Pt-SnO_2_/CP and Pt-SnO_2_@C/CP, respectively. The Pt loading was 0.15 mg cm^−2^ for both samples.

### 2.5. Physical Characterizations

Scan electron microscopy (SEM) (JSM-7100F, Tokyo, Japan), transmission electron microscopy (TEM, FEI TecnaiF30, Hillsboro, OR, USA) and energy-dispersive X-ray spectroscopy (EDX) were used to detect surface morphology, microstructures and element distribution.

The crystallinity and structure phases of the materials were determined by X-ray diffraction (XRD, Rigaku RINT2400, Tokyo, Japan) and Raman (LabRAMHR Evolution, Pairs, France) analysis.

Chemical states of the samples were examined by X-ray photoelectron spectroscopy (XPS, Kratos AXIS ULTRADLD, London, UK).

The contact angle was tested by a contact angle meter (Dataphysics OCA20, Stuttgart, Germany, water drop, 40 μL).

### 2.6. Electrochemical Characterization

Electrochemical measurements were carried out in a three-electrode electrochemical cell using a computer-controlled potentiostat (VMP2, Bio-logic Science Instruments, Paris, France) at room temperature (20 °C). The as-prepared Pt-SnO_2_/CP or Pt-SnO_2_@C/CP was used as the working electrode. A saturated calomel electrode (SCE) and a Pt plate (Purity, Shanghai Yue Magnetic Electronic Technology Co., Ltd., Shanghai, China) were used as the reference and counter electrodes, respectively. In this work, unless otherwise stated, all the potentials are expressed with respect to the reversible hydrogen electrode (RHE). The electrochemical performance tests were consistent with the single cell cathode test conditions [42,43].

Electrochemical impedance spectroscopy (EIS) was performed in the frequency range of 0.01–100 kHz in nitrogen and oxygen-saturated 0.5 M H_2_SO_4_ solution at an open circuit potential of 0.5, 0.4 and 0.3 V vs RHE. Cyclic voltammetry (CV) curves were obtained in a potential range of 0.05–1.2 V vs RHE at a scan rate of 50 mV s^−1^ in nitrogen-saturated 0.5 M H_2_SO_4_ solution. The electrochemical surface area (ECSA) value was determined from the integration of the hydrogen (desorption) region integrated from 0.05 to 0.4 V vs RHE. For the ADT testing, a total of 3000 cycles were performed between 0.6 to 1.2 V at a scan rate of 100 mV s^−1^ in nitrogen-saturated 0.5 M H_2_SO_4_ solution. The ECSA (in m^2^ g_pt_^−1^) was calculated for every 500 cycles using the following formula [44]

(1) $$S_{\mathrm{ECSA}}=\frac{Q_{\mathrm{Integraled}\;\mathrm{area}}\left(C\right)}{2.1\left(Cm^{-2}\right)\times {\mathrm{mass}}_{\mathrm{pt}}\left(\mathrm{mg}\right)}$$

where Q_Intergated area_ (C) and mass_Pt_ (mg) are the integrated charge and mass loading of Pt, respectively, and 2.1 (C m^−2^) corresponds to a monolayer hydrogen adsorption charge on polycrystalline Pt [11]. CV measurements were also performed in an oxygen-saturated 0.5 M H_2_SO_4_ solution to determine the peak potential for the oxygen reduction over the various electrocatalysts. In this study, commercial 40 wt.% Pt/C (Johnson Matthey Co., London, UK) was sprayed on a GDL as a baseline for comparison purpose. The loading of Pt was also 0.15 mg_pt_ cm^−2^.

## 3. Results and Discussion

**Figure 2:**
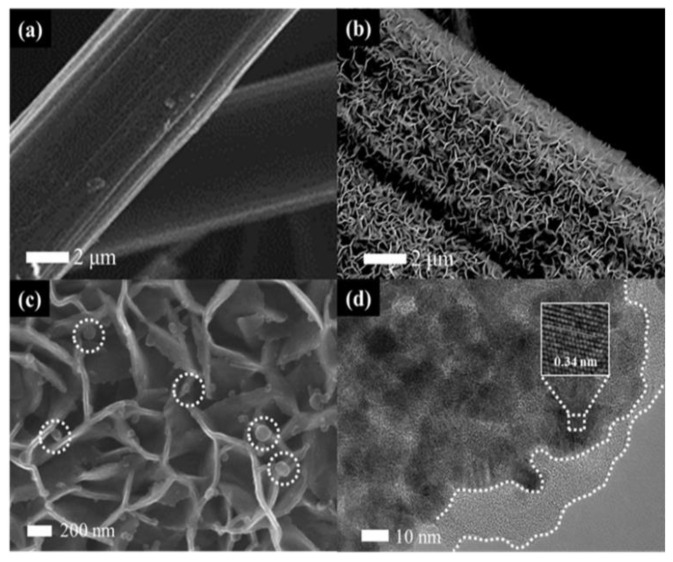
Scanning electron microscopy (SEM) images of: (**a**) carbon paper (CP); (**b**,**c**) SnO_2_@C/CP; and (**d**) transmission electron microscopy (TEM) image of SnO_2_@C/CP. The inset in (**d**) shows the high resolution transmission electron microscope (HRTEM) image of SnO_2_@C/CP. The circles in Figure 2c show the carbon spheres on SnO_2_@C/CP.

Figure 2 shows the morphology of CP and the prepared SnO_2_@C/CP OFA. It can be observed that SnO_2_@C flake arrays wrap around the surface of carbon fibres of CP. The overall shape of SnO_2_ did not change after the carbon coating, as shown in Figure S1, although high magnification SEM imaging (Figure 2c) shows that the flake thickness clearly increases. Compared with the support-free cathode, the flake support greatly enhances the surface area of the CP, thus improving the dispersion of the Pt catalyst. Small spherical particles (white circles in Figure 2c) are carbon spheres. The TEM image of SnO_2_@C (Figure 2d) shows that the SnO_2_ flakes are composed of particles and the carbon layer (with no lattice fringes) is coated on the surface of SnO_2_. The lattice fringes of SnO_2_ could be clearly observed in the inset of Figure 2d. The fringe spacing is 0.34 nm, consistent with the (110) plane of SnO_2_. The Bragg angle of (110) planes is 26.61°, although the CP support has a sharp diffraction peak at the same diffraction angle (Figure S2), thus making it difficult to distinguish the dominant diffraction peak of SnO_2_.

**Figure 3:**
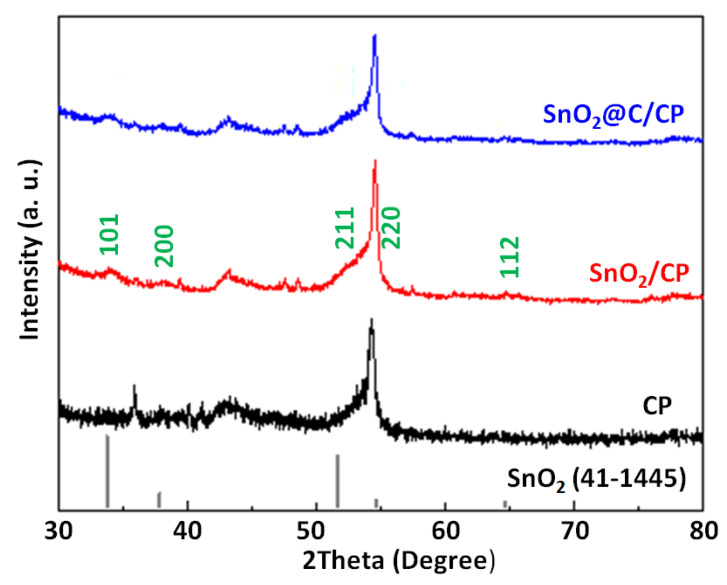
X-ray diffraction (XRD) patterns of CP, SnO_2_/CP and SnO_2_@C/CP.

Figure 3 shows the XRD patterns of CP, SnO_2_/CP and SnO_2_@C/CP for the scattering angles of 30° ≤ 2θ ≤ 80°. The peak at the scattering angle of 43.45° corresponds to the characteristic diffraction peak of the CP support. In addition, the peak located at 54° corresponds to the crystallitic graphite diffraction peak of the (004) plane on carbon paper [45], which was also reported by other researchers when CP was used as a support for active material [46]. Figure 3 also shows the main five peaks of SnO_2_, which can be indexed to SnO_2_ using the standard card (no. 41–1445). These peaks are assigned to a tetragonal rutile-structured SnO_2_, and indicate the formation of pure phase of SnO_2_. It is also clear that after the carbon coating, the characteristic peaks did not change.

**Figure 4:**
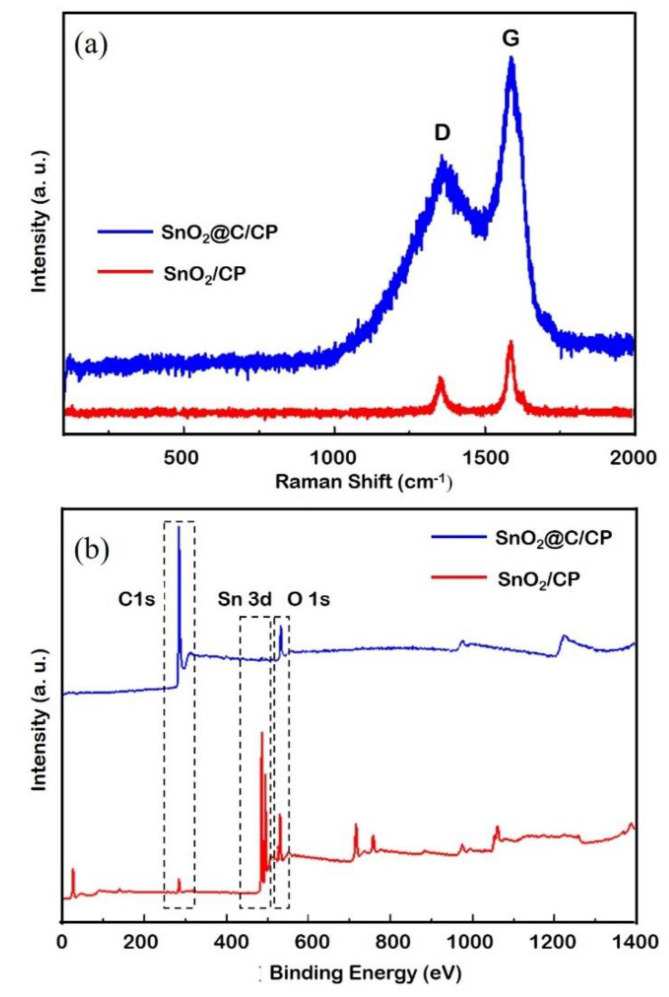
(**a**) Ramen spectra; and (**b**) X-ray photoelectron spectroscopy (XPS) spectra of SnO_2_/CP.

Figure 4a shows the Raman spectra of SnO_2_/CP and SnO_2_@C/CP. According to the literature [47], the Raman peaks located at 470 and 630 cm^−1^ are the characteristic peaks of SnO_2_, although these two peaks are not clearly observed. This is because the SnO_2_ array only exists on the surface of porous CP, and the thin SnO_2_ layer coated on the CP significantly reduces the sensitivity of the measurements. In addition, there are two sharp peaks located at 1353 and 1586 cm^−1^, which can be attributed to the D and G bands of the carbon, respectively. The relative intensity of the D and G bands (i.e., the areal peak intensity ratios of D band to G band) was calculated by the deconvolution of the spectral peaks using the Fityk software. The relative intensity of D and G bands is related to the amorphous carbon and defects. The relative intensity of D and G bands are increased from 0.53 to 0.64 after the carbon coating. The results imply that the carbon was coated on the SnO_2_ and formed a core-shell structure, which is consistent with the TEM result.

Figure 4b shows the XPS spectra of SnO_2_/CP and SnO_2_@C/CP flake arrays. A pair of peaks at binding energies of 486.3 and 495.3 eV, corresponding to the characteristic peaks of Sn^4+^, can be observed. The peaks at 531.1 and 284.85 eV correspond to the characteristic peak of O 1s and C 1s from SnO_2_, respectively. In the SnO_2_@C/CP spectrum, the intensity of Sn 3d and O 1s peaks decreases and that of C 1s increases, indicating that the carbon content in SnO_2_@C/CP increases and covers the surface of SnO_2_. Since the characteristic peak of Sn 3d can also be detected, the thickness of the carbon coating layer should be less than a few nanometers.

**Figure 5:**
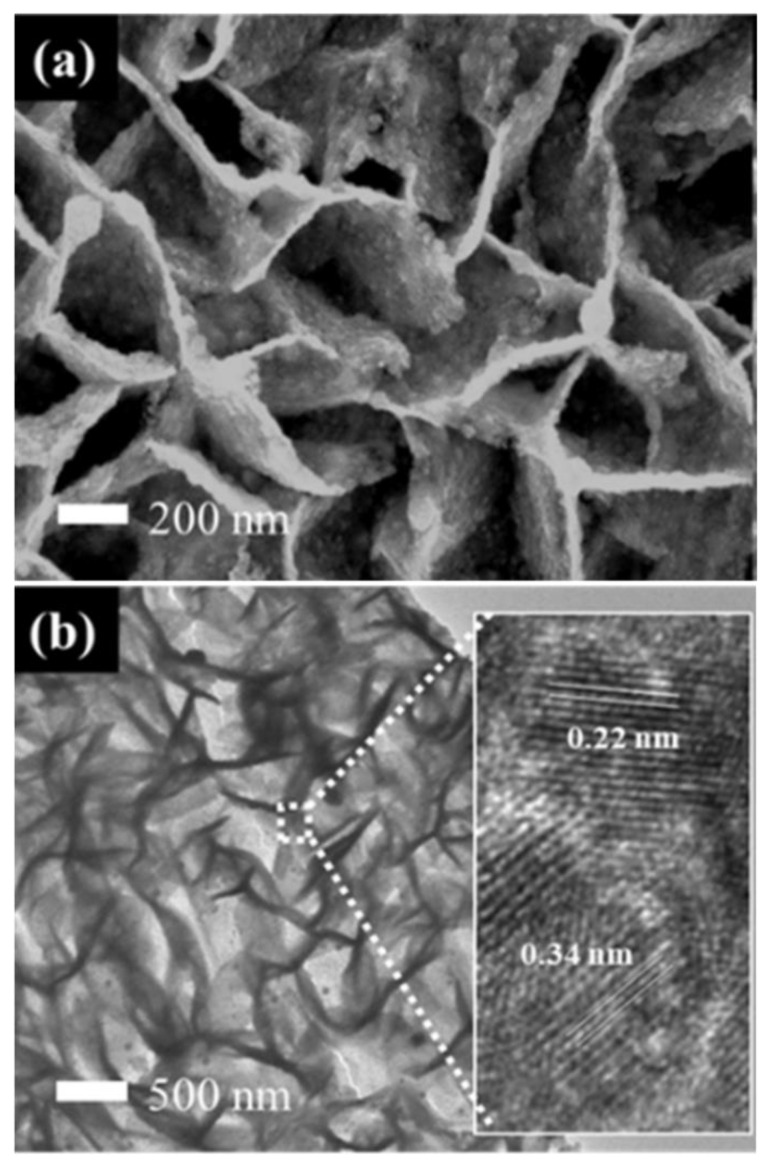
(**a**) SEM; and (**b**) TEM images of the Pt-SnO_2_@C/CP.

**Figure 6:**
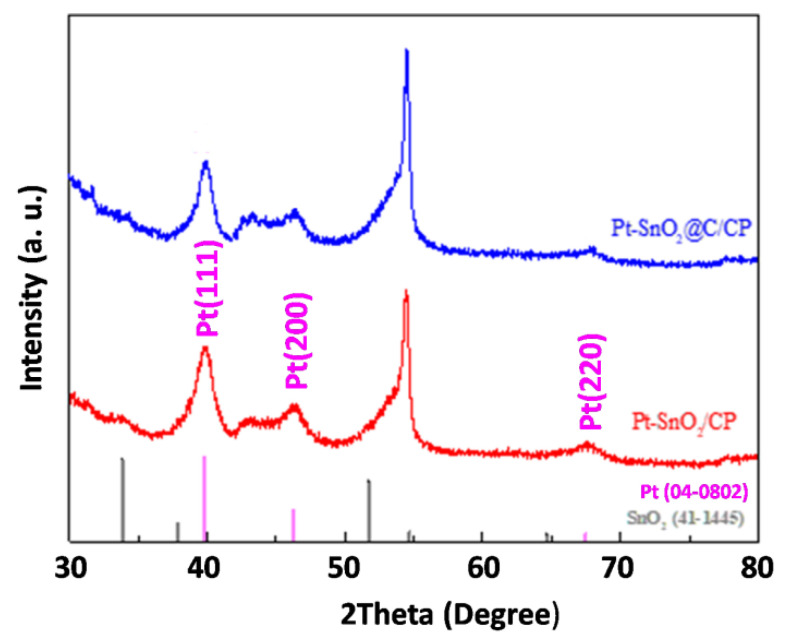
XRD patterns of Pt-SnO_2_/CP and Pt-SnO_2_@C/CP.

The SEM images of Pt-SnO_2_/CP and Pt-SnO_2_@C/CP are presented in Figure S3 and Figure 5a, respectively. It is clear that the deposited Pt nanoparticles exhibit different morphologies/sizes on the different supports. For the Pt-SnO_2_/CP sample, the spherical-shaped Pt grains of different particle sizes are embedded or mixed between the surfaces of the SnO_2_ flakes. This may be due to the fact that the surface layer of SnO_2_ lacks sufficient active sites for the even growth of Pt, thus leading to an uneven growth of Pt grains. Interestingly, on the surface of SnO_2_@C, there are almost no micron-sized Pt spheres, and the overall structure of the OFA does not change significantly after the Pt deposition. The Pt catalyst may cover the surface of the SnO_2_@C array as an aggregated Pt layer. TEM imaging (Figure 5b) shows that the overall morphology of Pt-SnO_2_@C is consistent with the SEM observation, and Pt is dispersed on the surface of SnO_2_@C as nanoparticles. The lattice fringes of Pt and SnO_2_ can be observed from the HRTEM image shown in Figure 5b (inset). The lattice fringe spacing of Pt is 0.22 nm, which corresponds to the Pt diffraction peak of the (111) plane in the XRD standard card. The EDS spectra (Figure S4) show that the SnO_2_/CP sample contained Sn, O and C, while significant Pt peaks were observed for the two Pt-based samples. It is thus clear that the non-uniform microspheres and uniform nanoparticles observed from the SEM images (i.e., Figure S3 and Figure 5a) belong to the Pt catalyst. The XRD patterns of SnO_2_- and SnO_2_@C-supported Pt catalysts are shown in Figure S5. A broad characteristic peak appears at a diffraction angle of ca. 40°, which corresponds to the diffraction peak of the Pt nanoparticles. For a more detailed analysis, partially magnified XRD patterns are presented in Figure 6. Diffraction peaks at scattering angles of 40, 46 and 67° are indexed to Pt (111), (200) and (220) planes, respectively, and are characteristic peaks of the faced-centered cubic crystalline structure of metallic Pt (no. 04–0802).

The presence of the carbon coating changes the morphology of the Pt, and increases the conductivity of the metal oxide. Figure S6a shows the Nyquist curves of SnO_2_/CP and SnO_2_@C/CP at open circuit potential in N_2_-saturated 0.5 M H_2_SO_4_ solution. The measured resistance value includes the resistance of the solution, each component and the internal resistance of the sample. The semiconducting SnO_2_ supported on CP has a resistance of 0.75 Ω cm^2^, while the SnO_2_@C/CP shows a resistance value of 0.64 Ω cm^2^, which is smaller than that of SnO_2_/CP. This implies that the carbon coating plays an important role in improving the electrical conductivity of SnO_2_ [38]. In addition to adequate electrical conductivity, the fuel cell cathode must also exhibit good hydrophobicity to avoid flooding during the operation at high current densities [48]. The prepared Pt-SnO_2_/CP and Pt-SnO_2_@C/CP with no hydrophobic treatment show sufficient hydrophobicity similar to the commercial Pt/C (Figure S7). The contact angle of commercial 40 wt.% Pt/C layer is 127.3°, while the SnO_2_/CP arrays are hydrophilic. After the SnO_2_/CP substrate was coated with a carbon layer, the hydrophobicity was improved and the contact angle reached 137.8°. This hydrophobicity is even significantly higher than that of the commercial Pt/C. This is probably because that the carbon layer obtained under the synthesis condition may contain the functional groups of long-chain carbon, which endowed the SnO_2_@C array surface with hydrophobic properties. After depositing Pt, the contact angle of the Pt-SnO_2_/CP and Pt-SnO_2_@C/CP reached 124.3 and 134.7°, respectively. These angles are close to, or exceed that of the commercial Pt/C. For the hydrophilic SnO_2_ support, the existence of Pt spheres may change the hydrophilicity. Different-sized Pt spheres grown on the surface of the SnO_2_ tend to increase the surface roughness, and as a result, the hydrophilicity of the surface also changes. For the Pt-SnO_2_@C/CP sample, the support material is hydrophobic and the Pt nanoparticles are uniformly distributed on the surface. This implies that the Pt surface may exhibit hydrophobicity.

**Figure 7:**
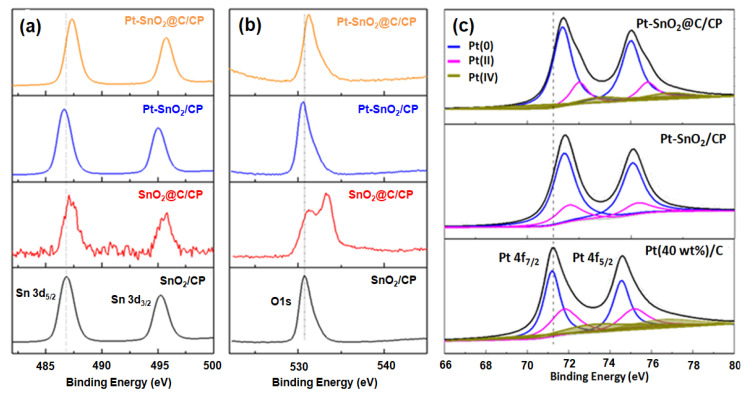
XPS spectra of: (**a**) Sn 3d; (**b**) O 1s; and (**c**) Pt 4f in SnO_2_/CP, SnO_2_@C/CP, Pt-SnO_2_/CP and Pt-SnO_2_@C/CP.

Figure 7a compares the XPS spectra of Sn 3d in different samples. The peaks located at 486.8 and 495.3 eV correspond to the binding energies of a Sn–O bond. Figure 7b shows the spectra of O1s, which correspond to that of Sn 3d in these samples. It is worth noting that there is one more peak at the binding energy of 533.4 eV for the SnO_2_@C/CP composite. This peak could be attributed to O1s of H_2_O derived from the aqueous mixture after Pt loading. Without the carbon coating, Pt spheres were directly loaded on the SnO_2_/CP, and the binding energy of Sn-O shifts towards lower binding energy levels. This was caused by the strong interaction between the Pt and SnO_2_ [49]. The positive shift of the Pt peak in Figure 7c also confirms this result. After the carbon coating, the binding energy of Sn-O shifts towards higher energy levels. According to the literature, this may be due to the formation of highly oxidized Sn-O bonds [47]. The characteristic peaks of the Pt-SnO_2_@C/CP have a similar binding energy at 487.3 and 495.8 eV to the SnO_2_@C/CP. Although the presence of the carbon layer the Pt peak still shifts towards higher binding energy, the interaction is still operative between Pt and SnO_2._ The synergistic effect between catalyst and metal oxide could result in higher catalytic activity [49]. Moreover, from the Pt peak fitting, there are three types of platinum: zero (0); bivalent (II); and tetravalent (IV). By observing the peak area, the Pt catalyst prepared by the room temperature reduction method has less Pt in the oxidation state, while the Pt (0) content has increased relatively, and thus the utilization rate of Pt has improved.

**Figure 8:**
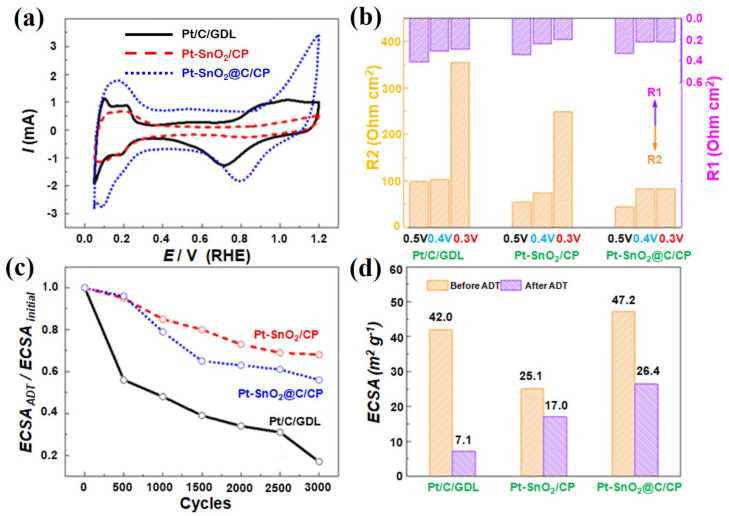
(**a**) Cyclic voltammetry (CV) curves; (**b**) impedance values at different potentials in O_2_ saturated solution; (**c**) degradation curves of electrochemical surface area (ECSA); and (**d**) ECSA before and after accelerated durability testing (ADT) for the Pt/C/GDL, Pt-SnO_2_/CP and Pt-SnO_2_@C/CP. R1 represents charge transfer resistance, while R2 represents mass transport resistance.

All electrochemical data collected for the as-synthesized catalysts were compared to the commercial Pt/C/GDL cathode layers. Figure S8 displays the CV plots for the various electrocatalysts from which ECSAs before and after ADT were determined and shown in Figure 8d. The Pt-SnO_2_/CP and Pt-SnO_2_@C/CP have an ECSA of 25.1 and 47.2 m^2^ g^−1^, respectively. Pt exists in the form of nanoparticles on the SnO_2_@C/CP, so the ECSA is higher. This ECSA is close to the commercial Pt/C catalyst, which has an ECSA of 42.0 m^2^ g^−1^. In addition, the as-developed Pt-SnO_2_@C/CP electrocatalyst outperformed many supported Pt catalysts reported in literature with a larger ECSA (Table S1, Supporting Information). However, the Pt-SnO_2_/CP shows significantly lower ECSA as the Pt spheres range from nanometers to micrometers.

Because the Pt catalyst grains were developed directly on the support, and it was difficult to strip the catalyst from the integrated electrode for oxygen reduction reaction (ORR) testing, the oxygen reduction activity was evaluated by the ORR peak potential in the CV curves, as shown in Figure 8a. For the commercial Pt/C, the peak potential of oxygen reduction is ca. 0.72 V, while it is ca. 0.83 V for the Pt-SnO_2_/CP, corresponding to a positive shift of ca. 110 mV. The Pt-SnO_2_@C/CP catalyst also shows a positive shift of ca. 80 mV in the peak potential, suggesting that the Pt SnO_2_@C/CP catalyst has improved the catalytic activity for the reduction of oxygen with respect to that of the Pt/C catalyst. The shift of the peak potential is probably attributed to the synergistic effect between the Pt catalyst and metal oxide [48].

Mass transport performance was evaluated by the EIS measurements at potentials of 0.5, 0.4 and 0.3 V in O_2_ saturated 0.5 M H_2_SO_4_ solution. In this potential range, the mass transfer resistance plays a dominant role in the potential losses [50]. Figure S6b–d show the Nyquist curves and Figure 8b displays the charge transfer and mass transport resistance values of the Pt/C/GDL, Pt-SnO_2_/CP and Pt-SnO_2_@C/CP in the above-mentioned potential range. The Pt-SnO_2_/CP and Pt-SnO_2_@C/CP show lower impedance compared to the commercial Pt/C, particularly in mass transport. As the value of potential decreased, the current increased, and the resistance for the charge transfer decreased, but the resistance for the mass transport increased. When the potential was lowered to 0.3 V, the resistance for the mass transport increased rapidly for the Pt/C/GDL and Pt-SnO_2_/CP, but interestingly, it also remained unchanged for the Pt-SnO_2_@C/CP. This is mainly attributed to the ordered structure and the hydrophobic surface, which enhanced the mass transport in the electrochemical process, and thus no significant increase in the mass transport resistance was observed [51].

Figure S8 also shows the CV curves of Pt/C/GD, Pt-SnO_2_/CP and Pt-SnO_2_@C/CP after various ADT cycles, from which the degradation trend and the values of ECSA after 3000 ADT cycles are shown in Figure 8c,d, respectively. As the cycling number increases, the ECSA gradually decreases. The ECSA of Pt/C decays rapidly, while the decay in the ECSA for the other two nanostructured catalysts is significantly slow. After 3000 ADT cycles, only 20% of the initial ECSA of the Pt/C remained, while the ECSA remained above 60% for the Pt-SnO_2_@C/CP flake array structure, and remained at about 70% for the Pt-SnO_2_/CP. The slower decay of ECSA of the Pt-loaded SnO_2_-based support arrays is partly due to the enhanced stability of the SnO_2_ support compared with the pure carbon support. It was reported that the combination of SnO_2_ and carbon could make catalysts more tolerant to high potential and rich oxygen environment at the cathode [36].

**Figure 9:**
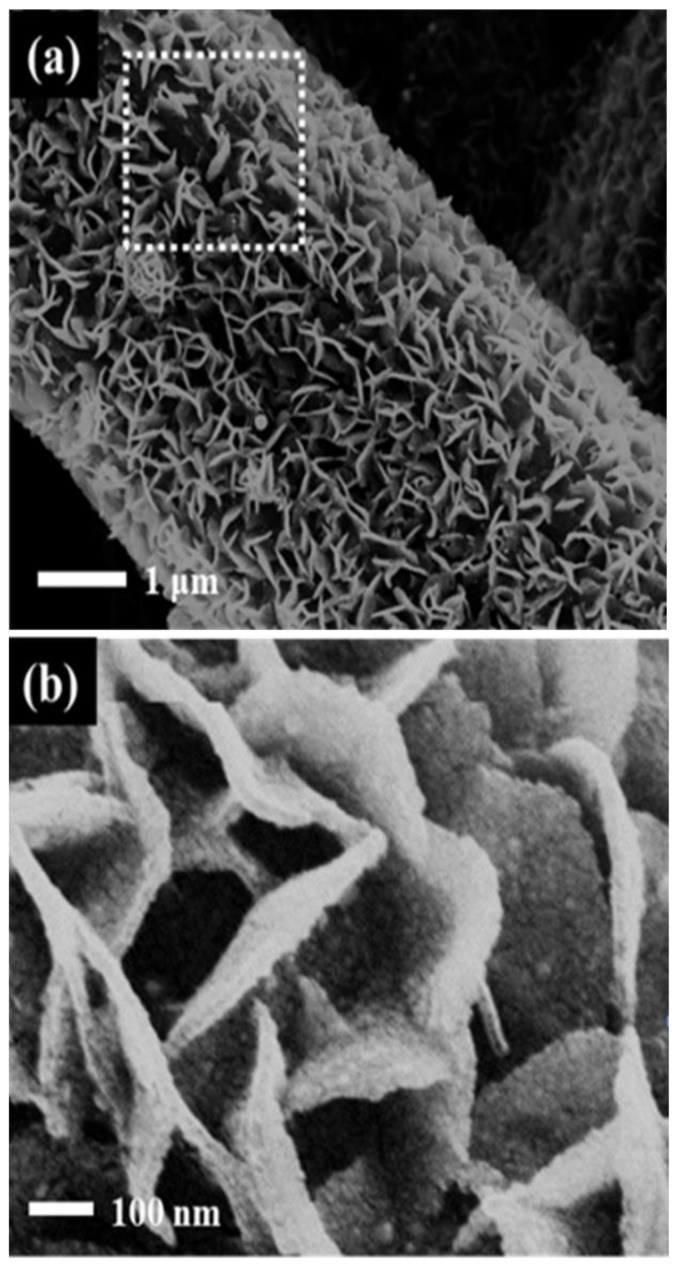
SEM images of the Pt-SnO_2_@C/CP after 3000 ADT cycles (**a**) Low magnification; (**b**). High magnification.

Figure S9 shows the SEM images of the Pt/C/GDL and Pt-SnO_2_/CP before and after ADT testing. The carbon support of the Pt/C catalysts suffers from corrosion when the catalyst is exposed to long-term stability test conditions of 0.6 to 1.2 V. Carbon as a support is easily oxidized at higher potentials (≥0.8 V), and corrosion proceeds via C + H_2_O →  CO_2_ + 4H^+^ + 4e^−^ [22]. As shown in Figure S9b, after the ADT testing, the corrosion of carbon caused the surface-supported Pt nanoparticles to migrate and aggregate, leading to the loss of an active surface area. Figure S9d shows the morphology of SnO_2_ support after the ADT tests. With no carbon coating, the SnO_2_ flake array is no longer clearly visible, and most of the SnO_2_ particles appear to have been aggregated. The Pt spheres, however, still exist between the SnO_2_ flakes, and SnO_2_ flakes appear to be coated on the surface of the Pt spheres. Due to the blocking effect of SnO_2_, Pt does not significantly migrate on the surface of SnO_2_, hence the decay of the active area is mainly due to the coating of SnO_2_ on the surface of Pt spheres, which reduces the surface-active sites of Pt. When the SnO_2_ is no longer changed, namely, the SnO_2_ coating on the surface of Pt spheres does not continue, the active area of the Pt catalyst becomes stable. Interestingly, for the Pt-SnO_2_@C/CP, the basic shape of the flaky array remains unchanged after the ATD testing. At the same time, the morphology of Pt does not show significant change, as observed from the SEM image shown in Figure 9. The difference before and after 3000 cycles is mainly due to a decrease in the flake array density. There is a large gap in the middle part of the rectangular frame where some parts of the support are lost, and as a result some Pt nanoparticles are also lost. Therefore, partial decay of the electrochemical activity occurs.

## 4. Conclusions

SnO_2_ flake array structures on CP were synthesized by hydrothermal method, and were further coated by carbon layer using the same method to form flake SnO_2_@C core-shell flake array. The Pt catalyst was supported on SnO_2_@C by a facile formic acid reduction method at room temperature. The presence of the carbon layer effectively enhanced the conductive conductivity and electrochemically active area. The improvement in the electrocatalytic activity mainly originated from the synergistic effects between SnO_2_ support and the carbon layer and Pt catalyst. The ordered array structure provided an effective three-phase reaction zone to accelerate the mass transport. Compared to the commercial Pt/C/GDL, the as-synthesized Pt-SnO_2_/CP and Pt-SnO_2_@C/CP showed lower electrochemical impedance in a low potential range. The ADT tests confirmed the better stability of the SnO_2_ support. This study further provides a facile and controllable method to prepare integrated ordered cathode array with high stability alongside enhanced electrocatalytic activity, which is believed to be helpful for preparing high-performance cathode for low-temperature fuel cells in the future.

## Supplementary Materials

The following are available online at https://www.mdpi.com/2079-4991/10/12/2412/s1, Figure S1: SEM images of the as-synthesized SnO_2_/CP with various magnifications, Figure S2. XRD patterns of CP, SnO_2_/CP and SnO_2_@C/CP, Figure S3. SEM images of the Pt-SnO_2_/CP with various magnifications, Figure S4. EDS spectra of SnO_2_/CP (a), Pt-SnO_2_/CP (b) and Pt-SnO_2_@C/CP (c), Figure S5. XRD patterns of Pt-SnO_2_/CP and Pt-SnO_2_@C/CP, Figure S6. Nyqiust plots of SnO_2_/CP and SnO_2_@C/CP (a) recorded at open circuit potential in N_2_ saturated 0.5 M H_2_SO_4_ solution, Nyqiust plots of Pt/C/GDL (b), Pt-SnO_2_/CP (c) and Pt-SnO_2_@C/CP (d) at the potential of 0.5 V, 0.4 V and 0.3 V in O_2_-saturated 0.5 M H_2_SO_4_ solution, Figure S7. Contact angles of CP (a), SnO_2_/CP (b), SnO_2_@C/CP (c), 40 wt%Pt/C/GDL (d), Pt-SnO_2_/CP (e) and Pt- SnO_2_@C/CP (f), Figure S8. CV curves recorded in N_2_-saturated 0.5 M H_2_SO_4_ for the Pt/C/GDL (a), Pt-SnO_2_/CP (b) and Pt-SnO_2_@C/CP (c) during 3000 ADT cycles, Table S1. Comparison of the ESCA of the Pt-SnO_2_@C/CP with that reported in literature in acidic solutions, Figure S9. SEM images of the Pt/C/GDL (a, b) and the Pt-SnO_2_/CP (c, d) before and after ADT.
