# Supplementary material for: Ordered SnO2@C Flake Array as Catalyst Support for Improved Electrocatalytic Activity and Cathode Durability in PEMFCs
Source: Nanomaterials (Basel). 2020 Dec 2;10(12):2412. doi: 10.3390/nano10122412 (PMC7761613; doi:10.3390/nano10122412)
Supplement: Supplementary file 1 [file nanomaterials-10-02412-s001.pdf]

Supplementary Materials

Ordered SnO_2@C Flake Array as Catalyst Support for Improved Electrocatalytic Activity and Cathode Durability in PEMFCs

Zhaoyi Yang [1,2], Ming Chen [1,2], Baizeng Fang [3,*] and Gaoyang Liu [1,2,*]

[1] School of Metallurgical and Ecological Engineering, University of Science and Technology Beijing, 30 College Road, Beijing, 100083, China; zhaoyiyangustb@163.com (Z.Y.); chenm3@sustech.edu.cn (M.C.)
[2] Beijing Key Laboratory for Magneto-Photoelectrical Composite and Interface Science, University of Science and Technology Beijing, 30 College Road, Beijing, 100083, China
[3] Department of Chemical and Biological Engineering, University of British Columbia, 2360 East Mall, Vancouver, British Columbia, V6T 1Z3, Canada
* Correspondence: bfang@chbe.ubc.ca (B.F.); liugy@ustb.edu.cn (G.L.)

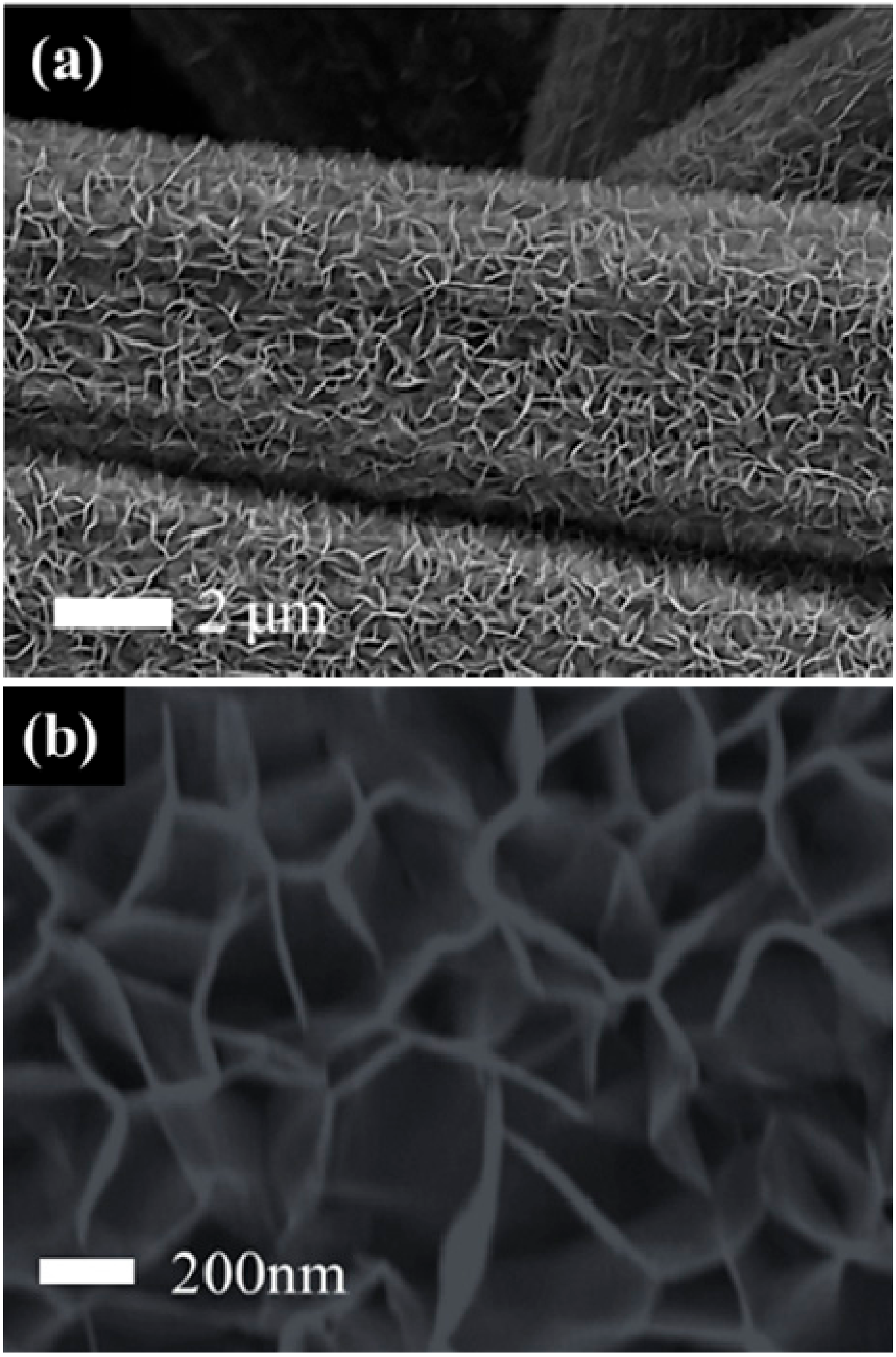

Figure S1. SEM images of the as-synthesized SnO_2/CP with various magnifications: (a) low magnification, (b) high magnification.

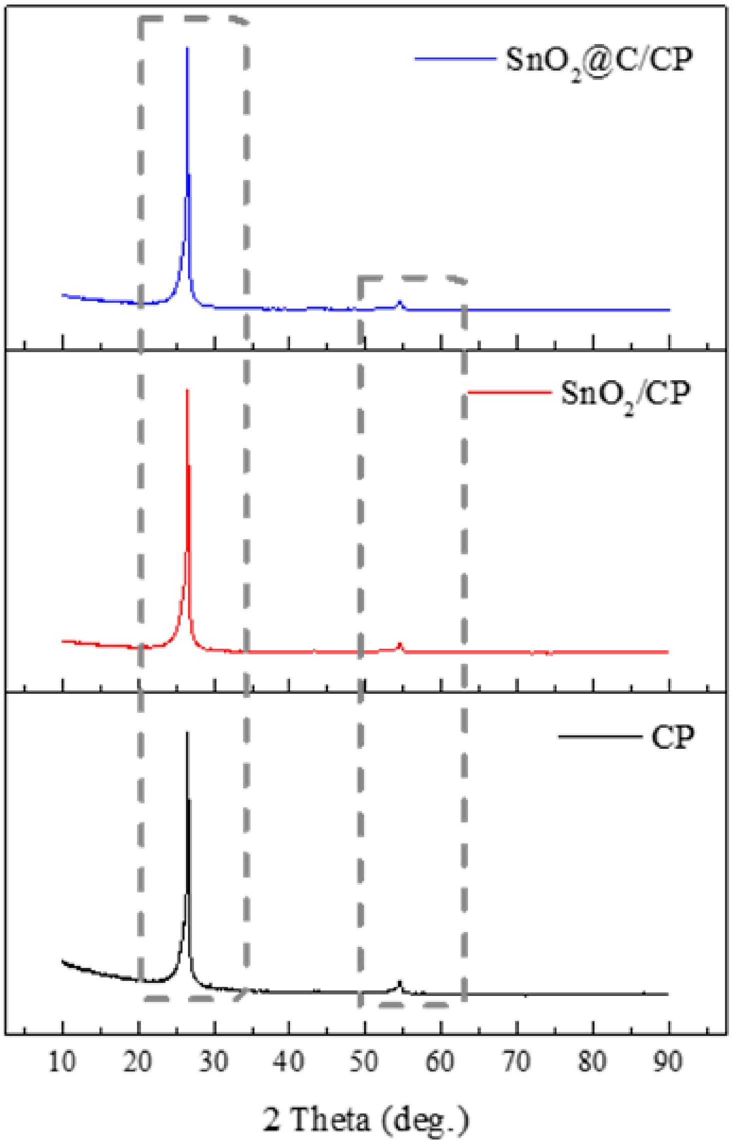

Figure S2. XRD patterns of CP, SnO_2/CP and SnO_2@C/CP.

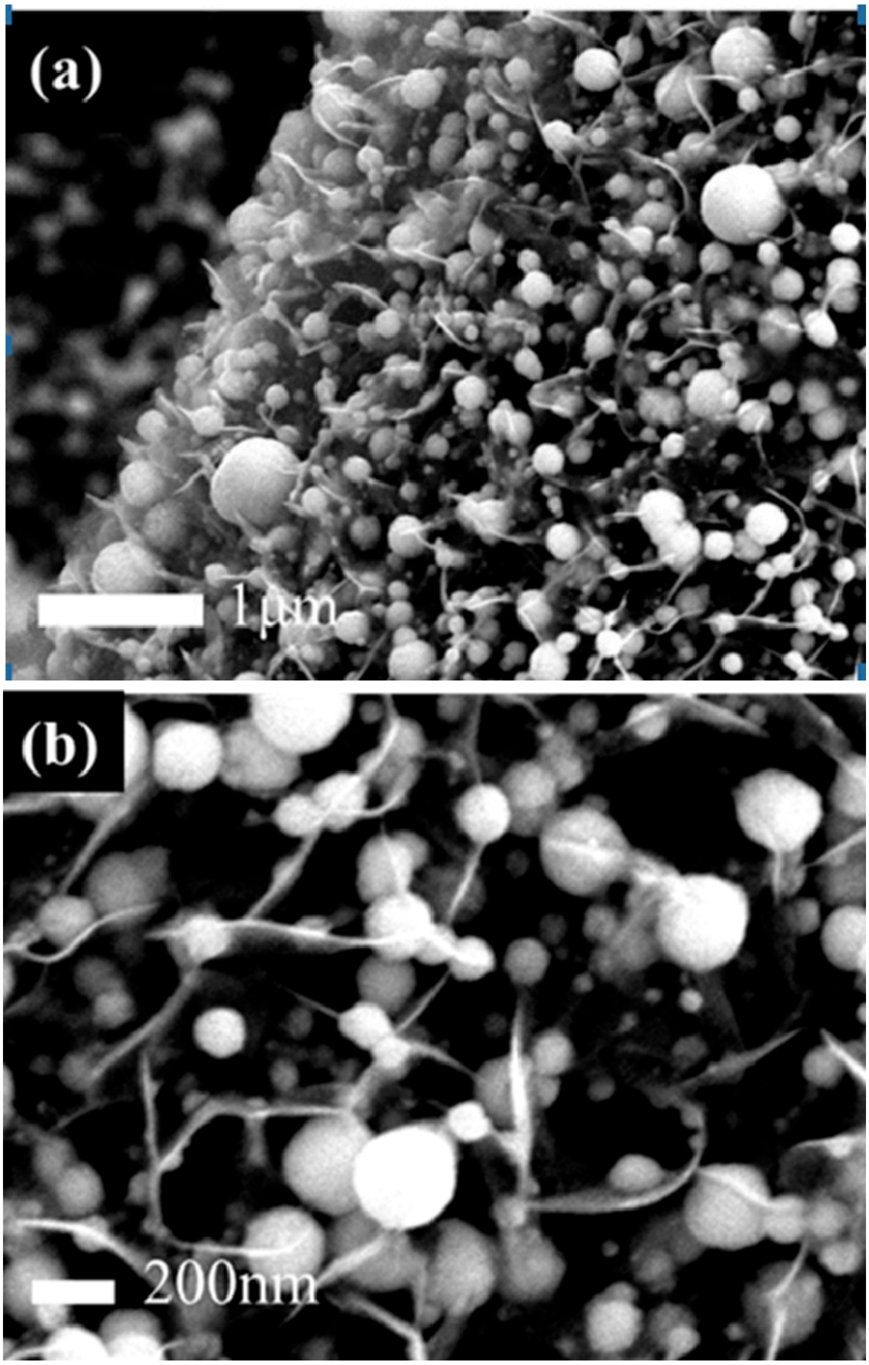

Figure S3. SEM images of the Pt-SnO_2/CP with various magnifications: (a) low magnification, (b) high magnification.

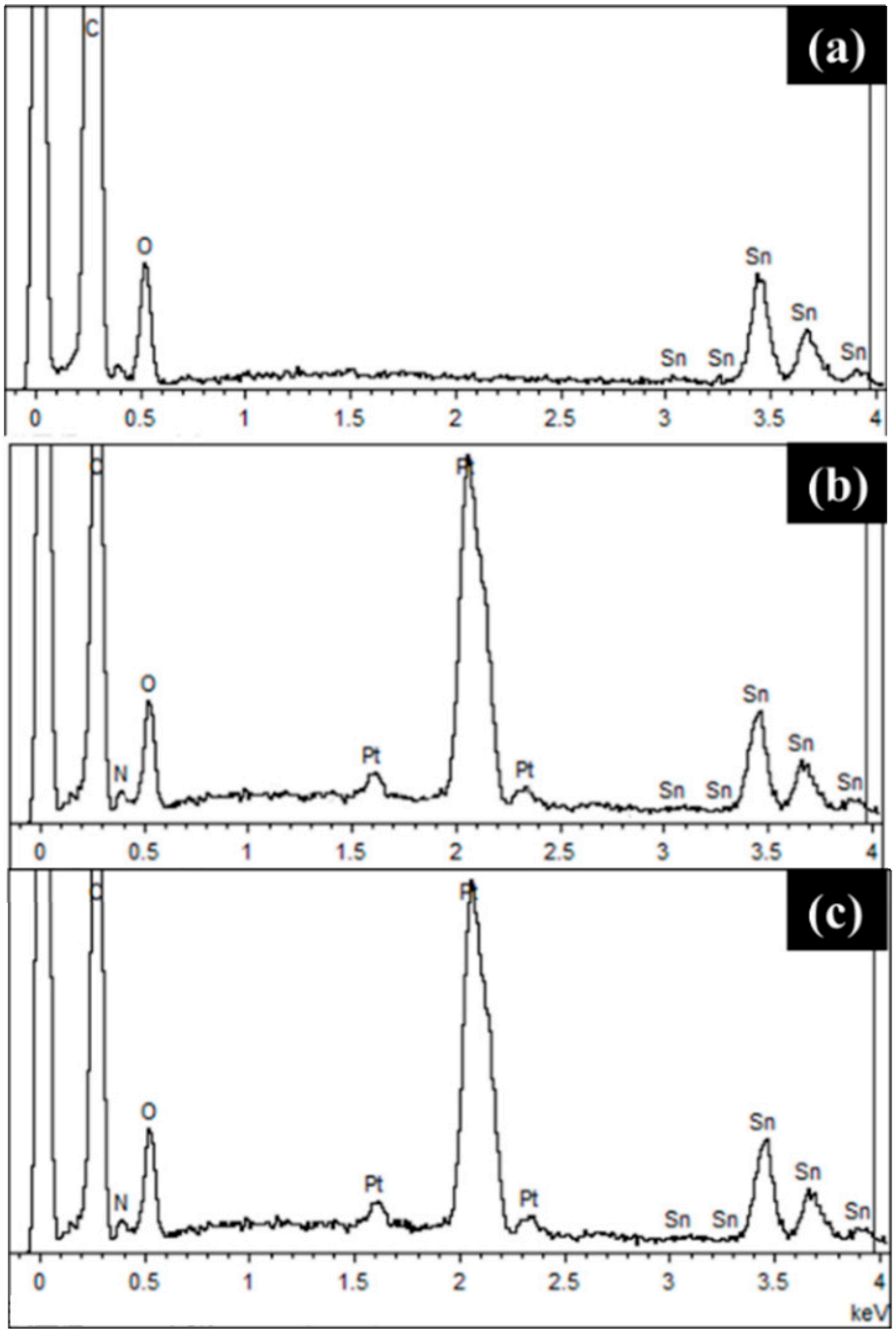

Figure S4. EDS spectra of SnO_2/CP (a), Pt-SnO_2/CP (b) and Pt-SnO_2@C/CP (c).

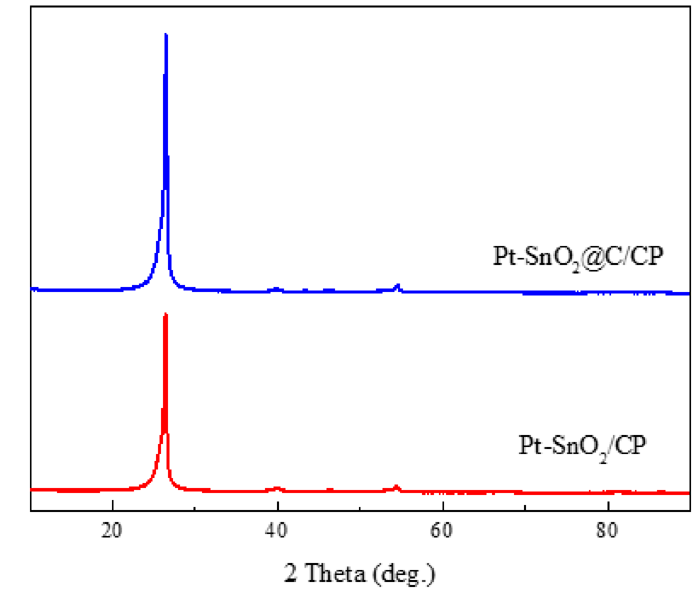

Figure S5. XRD patterns of Pt-SnO_2/CP and Pt-SnO_2@C/CP.

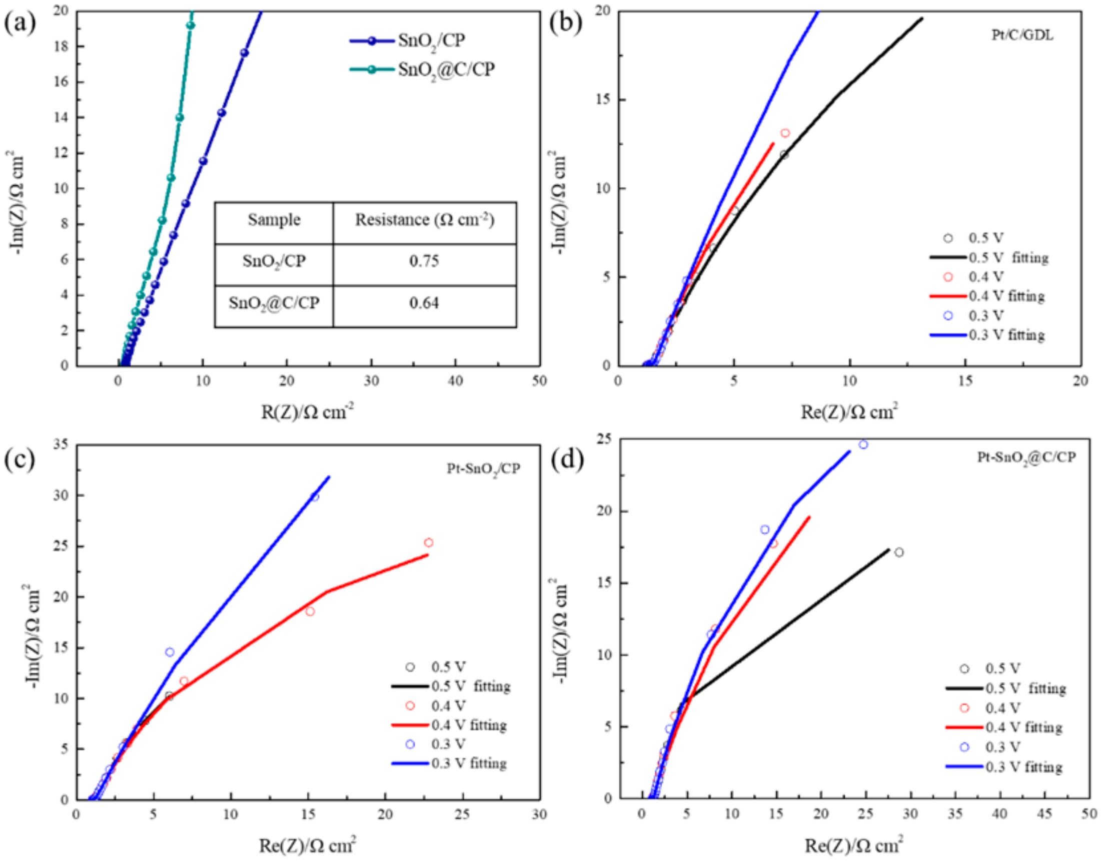

Figure S6. Nyqiust plots of SnO_2/CP and SnO_2@C/CP (a) recorded at open circuit potential in N_2 saturated 0.5 M H_2SO_4 solution. Nyqiust plots of Pt/C/GDL (b), Pt-SnO_2/CP (c) and Pt-SnO_2@C/CP (d) at the potential of 0.5 V, 0.4 V and 0.3 V in O_2-saturated 0.5 M H_2SO_4 solution.

Note: The EIS analysis was carried out based on the literature (K. Su, et al. Ionomer content effects on the electrocatalyst layer with in-situ grown Pt nanowires in PEMFCs, Int. J. Hydrogen Energ. 2014, 39, 3219-3225; Heijne et al, Identifying charge and mass transfer resistances of an oxygen reducing biocathode. Energy Environ. Sci., 2011, 4, 5035). Both the charge transfer resistance and the mass transport resistance, which are usually determined by the semi-circle and linear response in Nyquist plot, were obtained by fitting the experimental data using ZSimpWin software. The equivalent electrical circle shown below was selected to fit the impedance data.

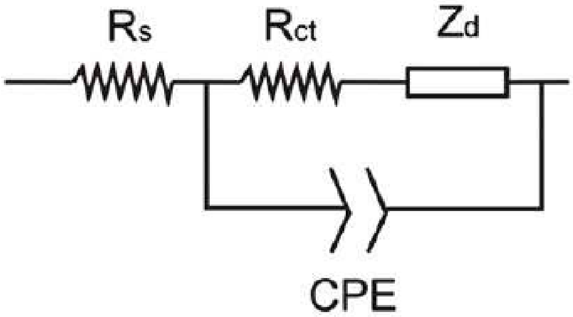

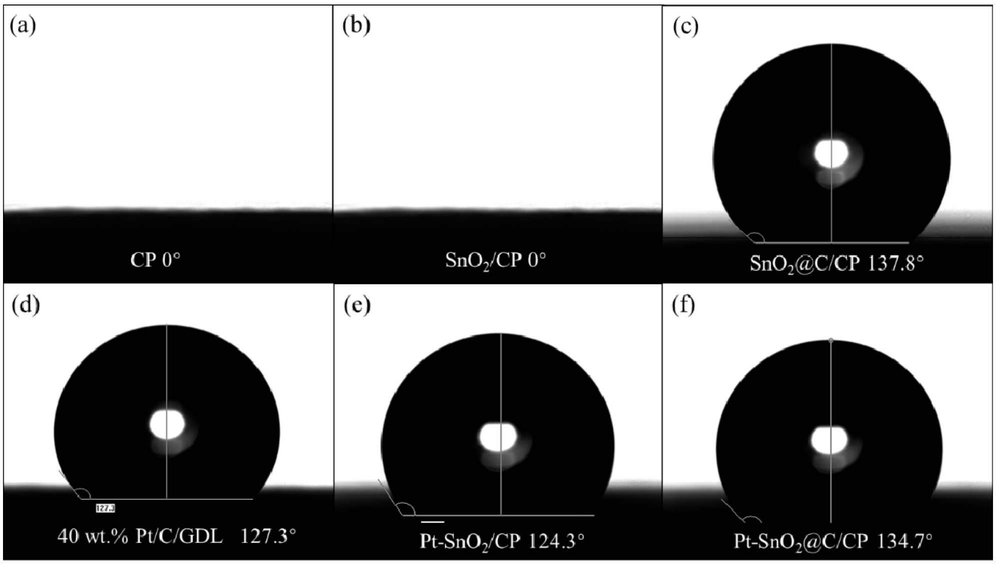

Figure S7. Contact angles of CP (a), SnO_2/CP (b), SnO_2@C/CP (c), 40 wt%Pt/C/GDL (d), Pt-SnO_2/CP (e) and Pt- SnO_2@C/CP (f).

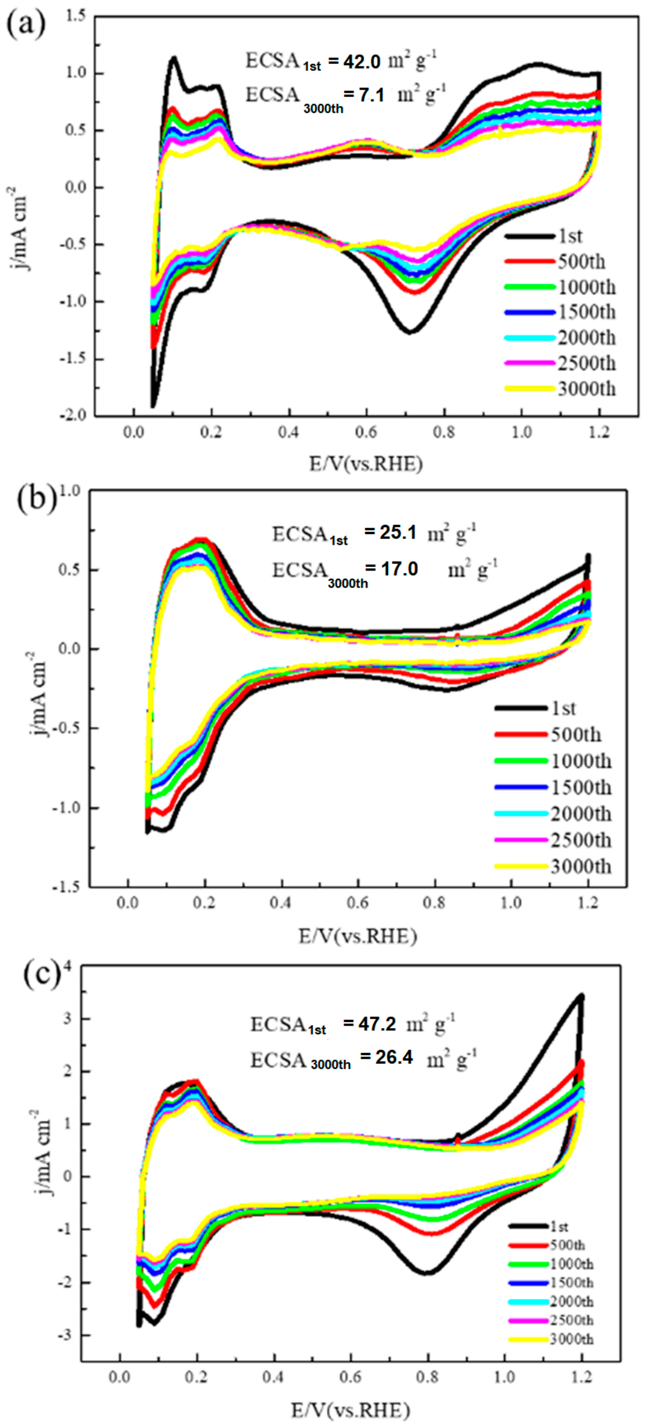

Figure S8. CV curves recorded in N_2-saturated 0.5 M H_2SO_4 for the Pt/C/GDL (a), Pt-SnO_2/CP (b) and Pt-SnO_2@C/CP (c) during 3,000 ADT cycles.

Table S1. Comparison of the ESCA of the Pt-SnO_2@C/CP with that reported in literature in acidic solutions.

Catalyst	Substrate	Electrolyte	$ECSA_{initial}$ $m^2\ g^{-1}$	Reference
Pt-SnO_2@C OFA	Carbon paper	0.5 M H_2SO_4	47.2	This work
Pt/ATO nanofiber	Graphite Carbon	0.5 M H_2SO_4	33	Electrocatalysis, (2019), 10:262–271
Pt/ATO nano particles	Graphite Carbon	0.5 M H_2SO_4	26	Electrocatalysis, (2019), 10:262–271
formic-Pt/ATO	Glassy Carbon	0.1 M $HClO_4$	28	ACS Appllied Energy Materials, (2020), 3(6): 5774–5783
ethylene--Pt/ATO	Glassy Carbon	0.1 M $HClO_4$	31	ACS Appllied Energy Materials, (2020), 3(6): 5774–5783
Pt/SnO_2	Glassy Carbon	0.1 M $HClO_4$	~16	Electrochem. Solid-State Letters, (2009), 12 B119
Pt–SnO_2(400)/CN_x	Glassy Carbon	0.5 M H_2SO_4	52	Journal of Power Sources, (2013), 238(15): 144-149

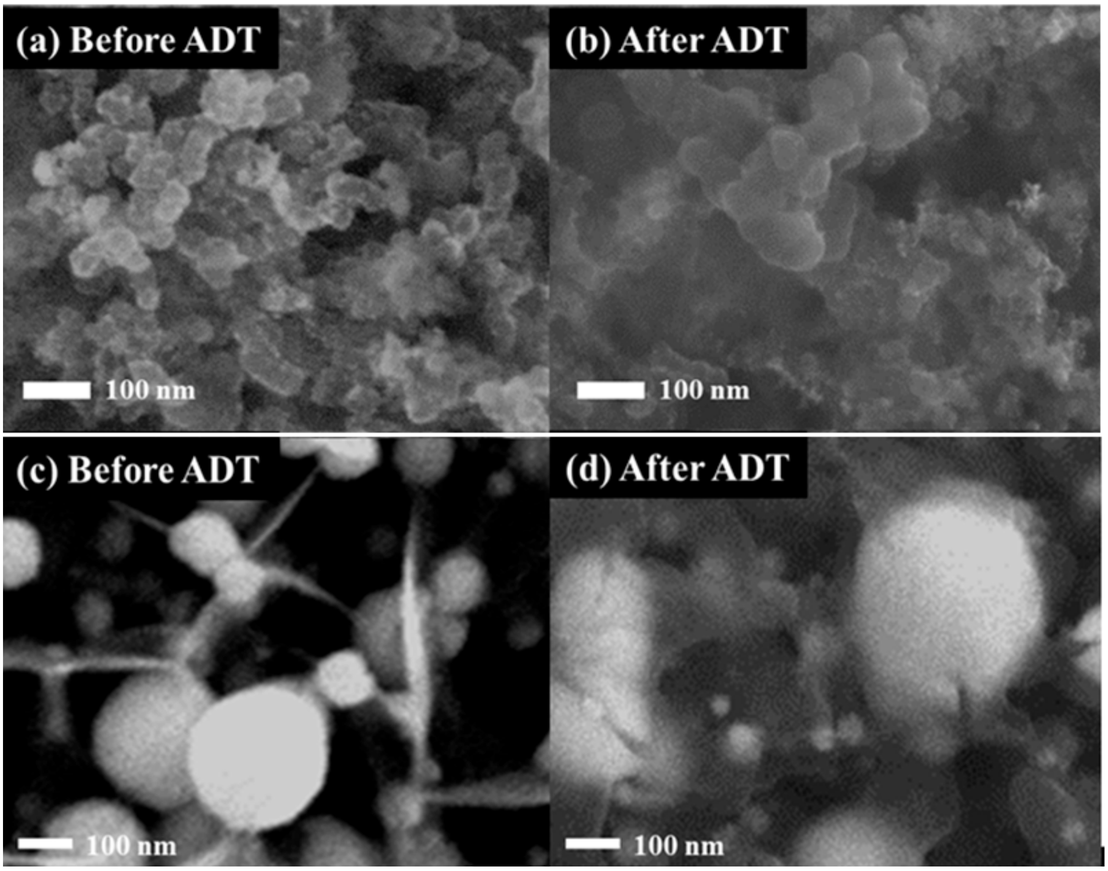

Figure S9. SEM images of the Pt/C/GDL (a, b) and the Pt-SnO_2/CP (c, d) before and after ADT.
